# Enhanced metabolomic predictions using concept drift analysis: identification and correction of confounding factors

**DOI:** 10.1093/bioadv/vbaf073

**Published:** 2025-04-04

**Authors:** Jana Schwarzerova, Dominika Olesova, Katerina Jureckova, Ales Kvasnicka, Ales Kostoval, David Friedecky, Jiri Sekora, Jitka Pomenkova, Valentyna Provaznik, Lubos Popelinsky, Wolfram Weckwerth

**Affiliations:** Department of Functional and Evolutionary Ecology, Molecular Systems Biology (MOSYS), University of Vienna, Vienna 1010, Austria; Department of Biomedical Engineering, Faculty of Electrical Engineering and Communication, Brno University of Technology, Brno 616 00, Czech Republic; Department of Molecular and Clinical Pathology and Medical Genetics, University Hospital Ostrava, Ostrava 708 00, Czech Republic; Institute of Experimental Endocrinology, Biomedical Research Center, Slovak Academy of Sciences, Bratislava 845 05, Slovak Republic; Institute of Neuroimmunology, Slovak Academy of Sciences, Bratislava 845 05, Slovak Republic; Department of Biomedical Engineering, Faculty of Electrical Engineering and Communication, Brno University of Technology, Brno 616 00, Czech Republic; Laboratory for Inherited Metabolic Disorders, Department of Clinical Biochemistry, University Hospital Olomouc, Olomouc 779 00, Czech Republic; Faculty of Medicine and Dentistry, Palacký University Olomouc, Olomouc 779 00, Czech Republic; Department of Biomedical Engineering, Faculty of Electrical Engineering and Communication, Brno University of Technology, Brno 616 00, Czech Republic; Laboratory for Inherited Metabolic Disorders, Department of Clinical Biochemistry, University Hospital Olomouc, Olomouc 779 00, Czech Republic; Faculty of Medicine and Dentistry, Palacký University Olomouc, Olomouc 779 00, Czech Republic; Department of Biomedical Engineering, Faculty of Electrical Engineering and Communication, Brno University of Technology, Brno 616 00, Czech Republic; Department of Radio Electronics, Faculty of Electrical Engineering and Communication, Brno University of Technology, Brno 616 00, Czech Republic; Department of Biomedical Engineering, Faculty of Electrical Engineering and Communication, Brno University of Technology, Brno 616 00, Czech Republic; Department of Physiology, Faculty of Medicine, Masaryk University, Brno 625 00, Czech Republic; Faculty of Informatics, Masaryk University, Brno 602 00, Czech Republic; Department of Functional and Evolutionary Ecology, Molecular Systems Biology (MOSYS), University of Vienna, Vienna 1010, Austria; Vienna Metabolomics Center (VIME), University of Vienna, Vienna 1010, Austria

## Abstract

**Motivation:**

The increasing use of big data and optimized prediction methods in metabolomics requires techniques aligned with biological assumptions to improve early symptom diagnosis. One major challenge in predictive data analysis is handling confounding factors—variables influencing predictions but not directly included in the analysis.

**Results:**

Detecting and correcting confounding factors enhances prediction accuracy, reducing false negatives that contribute to diagnostic errors. This study reviews concept drift detection methods in metabolomic predictions and selects the most appropriate ones. We introduce a new implementation of concept drift analysis in predictive classifiers using metabolomics data. Known confounding factors were confirmed, validating our approach and aligning it with conventional methods. Additionally, we identified potential confounding factors that may influence biomarker analysis, which could introduce bias and impact model performance.

**Availability and implementation:**

Based on biological assumptions supported by detected concept drift, these confounding factors were incorporated into correction of prediction algorithms to enhance their accuracy. The proposed methodology has been implemented in Semi-Automated Pipeline using Concept Drift Analysis for improving Metabolomic Predictions (SAPCDAMP), an open-source workflow available at https://github.com/JanaSchwarzerova/SAPCDAMP.

## 1 Introduction

The Advances in computational science, particularly the integration of artificial intelligence, machine learning (ML), and deep learning (DL), are transforming the processing and utilizing of large-scale biomedical datasets. These technologies and enable personalized treatments by leveraging patient-specific data. However, a major challenge in modelling biological processes lies in balancing the abundance of generalized data with the specificity required for individual predictions. It is nearly impossible to train predictive models on generalized datasets and expect accurate, highly specific predictions for a particular patient group. Conversely, focusing exclusively on narrowly defined datasets results in insufficient training, testing, and validation data. This trade-off remains a key bottleneck in developing precise predictive tools, not only for clinical applications but also for non-medical field, such as plant breeding programs aimed to addressing climate challenges.

This phenomenon in ML is known as concept drift (Webb *et al.* 2016). In the context of biomedical data, the metabolome represents one of the molecular networks that undergo significant changes over time, reflecting various life cycles, illnesses or stress responses. Leveraging metabolomic analysis offers a powerful tool for preventive healthcare, pharmaceutical development, and even ecological engineering. The prediction of disease progression, treatment response monitoring, and early diagnostics are major topics in biomedical research. Currently, metabolomics-based screening, biomarkers, and surrogate markers are being incorporated into clinical trials and drug development. This creates new challenges for optimization and validation of *in silico* methods (Jeanquartier *et al.* 2016), that need to be more robust, and produce reliable results. Due to the complexity, regulation and dynamic behaviour of metabolic pathways, prediction models still suffer from a very low success rate in metabolomics (Cohen and Harel 2007, Kugler *et al.* 2010). All prediction algorithms and ML approaches work best on static data (Žliobaitė *et al.* 2016) by assuming that the data behaviour is constant, however, in human, animal, plant, and microbial metabolism the opposite is the case, biological systems are subjects of perpetual metabolic turnover (Rattray *et al.* 2017). Despite vast knowledge of animal and plant biochemistry, there are still many gaps that are not fully elucidated (Huangyang and Simon 2018, Ivanisevic and Thomas 2018, Simsek *et al.* 2018). It is possible that we will not be able to describe all of them in detail, as the complexity of a living system increases the computational complexity (Dubey *et al.* 2020).

However, by accounting for the complex behaviour of biological systems and relying on biologically validated assumptions, we can effectively apply and optimize modern computational methods, including the implementation and refinement of concept drift in predictive models. When applied to metabolomic and biological datasets, two distinct challenges emerge: data cleaning and the removal of outliers (Fernández Pierna *et al. * 2002), and the broader issue of determining whether it is appropriate to harmonize measurements across different batches due to potential batch effects. Advanced ML methods, which focus on training classifiers based on patterns and relationships within the data rather than specific metabolite values, require these challenges to be reconsidered. In particular, it is essential to address how data drift—caused by outliers or other factors—impacts the overall significance and reliability of predictive models. Unfortunately, these blind spots contribute to the low predictive power of metabolomic prediction models. They are typically represented by confounding factors in input data which can be revealed using concept drift analysis based on concept drift detection (CDD).

### 1.1 Unlocking insights into concept drift from a computational biology perspective

Concept drift is a particularly prominent issue in biomedical datasets, where biological dynamics—such as metabolic adaptations, environmental changes, or microbial resistance to antibiotics—are in constant flux. These ongoing shifts disrupt ML models by invalidating previously learned patterns, leading to potential inaccuracies in detection and prediction tasks.

It is equally important to distinguish between concept drift and data drift, as they present unique challenges in ML. While often related, the two phenomena differ fundamentally in their impact. Data drift occurs when there are changes in the statistical properties of input data, such as shifts in feature distributions or measurement conditions over time. On the other hand, concept drift involves a change in the relationship between input features and the target variable, altering the core meaning of the predictive task. For example, in biomedical applications, data drift might manifest due to variations in experimental setups, sensor recalibrations, or differences in batch processing. In contrast, concept drift could arise from more fundamental changes, such as the emergence of new disease phenotypes or shifts in how biomarkers correlate with clinical outcomes. While data drift challenges models by affecting the inputs, concept drift necessitates re-evaluating the interpretation and predictive value of those inputs. To provide a comprehensive understanding of the differences and implications of these phenomena, Table 1 summarizes their definitions, examples, impacts, detection techniques, and potential responses.

**Table 1.** Overview of the comparison between data drift and concept drift.

Aspect	Data drift	Concept drift
*Definition*	Changes in the statistical properties of input data	Shifts in the relationship between input features and the target variable, altering the meaning of predictions
*Impact*	Primarily affects input data distributions; may degrade model performance if not accounted for.	Requires retraining or adjusting models to account for new input-output relationships
*Detection Techniques*	Statistical tests, such as monitoring feature distributions over time	Monitoring model error rates, decision boundaries, or predictive inconsistencies
*Response*	Data pre-processing, re-sampling, updating data pipelines, or retraining the model with updated data.	Retraining the model, redesigning features, incorporating online learning, or using adaptive models
*Example 1*	Variations in experimental setups, such as changes in instrument calibration	Emergence of new disease phenotypes altering the association between symptoms and diagnosis
*Example 2*	Differences in environmental conditions affecting data collection	Evolving microbial resistance to antibiotics changing treatment efficacy predictions
*Example 3*	Batch effects in metabolomics experiments	Shifts in biomarker/marker relevance for predicting disease severity

While concept drift is frequently discussed in ML literature, with a primary focus on enhancing predictive performance metrics (e.g. AUC, F1-score), it is critical to recognize that metabolomics research is not primarily centred on building predictive algorithms as an end goal. Instead, predictive modelling in this domain serves as a means to support broader biological insights and interpretations. CDD, therefore, holds significance beyond improving classifier accuracy, extending into areas essential for maintaining the integrity and reproducibility of metabolomic studies. Incorporating CDD into metabolomics workflows has the potential to facilitate standardization and harmonization across studies, particularly in collaborative research projects involving multiple labs or longitudinal studies spanning extended time periods. By providing a robust mechanism to monitor and correct for drift, these methods could enhance data comparability and reproducibility, addressing one of the key challenges in metabolomics research.

However, the current detection methods are built on evaluating statistical features such as the mean and standard deviation of the samples, error rates, or some other metrics of the base models, e.g. it is designed for ML models based on classification tasks such as decision tree learning as in the Ultra-Fast Forest of Trees system (João Gama *et al.* 2005; Schwarzerova *et al.* 2021) where CDD uses Naïve Bayes classifier. This classifier offers an attractive intuitive solution based on statistical theory, which states that if the distribution is stationary, the online error of naïve-Bayes will decrease or stay the same. Otherwise, the detected increasing error rate represents the concept drift phenomenon. The classifier needs to upgrade decision-making criteria. Even though the CDD cannot avoid false positive detection, it can help to improve prediction models such as those used for prediction of growth rates and metabolite concentrations, which have considerable potential in early disease detection (Trairatphisan *et al.* 2016, Schwarzerova *et al.* 2021, 2022).

The most used methods for CDD includes algorithms such as the Drift Detection Method (DDM) (Costa *et al.* 2018, João Gama *et al.* 2005) and early drift detection method (EDDM) (Baena-García *et al.* 2006). DDM and EDDM are further extended by predictive algorithms related to spam email detectors, or for financial stock market regression problems. Other algorithms that uses to CDD are e.g.: statistical test of equal proportions to detection (Kyosuke and Yamauchi 2007), Equal Means Z-Test Concept Drift Detector (Cabral and Barros 2020); Weighted Statistical Process Control (EWMA) (Roberts 2000); Concept Length Shortening (RDDM) (Barros *et al.* 2017), and the Hoeffding Drift Detection Method (HDDM) (Frías-Blanco *et al.* 2015). Our previous studies (Schwarzerova *et al.* 2021, 2022) showed the best candidates within the CDD in metabolomic prediction so far are still DDM and EDDM.

For more complex and evolving datasets, unsupervised approaches, such as clustering-based or distribution-monitoring methods, offer advantages. These methods are particularly effective when time labels are absent or continuous monitoring of data streams is necessary. By addressing both data drift and concept drift in tandem, predictive models can be better adapted to the intricate and dynamic nature of biomedical datasets, ensuring more accurate and reliable outcomes in real-world applications.

In this study, we compare individual CDD methods on different metabolomic training and test datasets to reveal gender differences. Due to the analysis, we are able to identify confounding factors influencing the prediction, however, these factors are not considered in the prediction model. After revealing these confounding factors, it is possible to correct the metabolomic predictions in order to obtain more accurate models. The concept drift in predictive models can be revised using concept drift correction that is based on novel biological relationships allowing for improved prediction models to facilitate the outcome of medical (Ip *et al.* 1991, Schwarzerova *et al.* 2022) and pharmaceutical studies.

## 2 Methods

**Figure 1.**
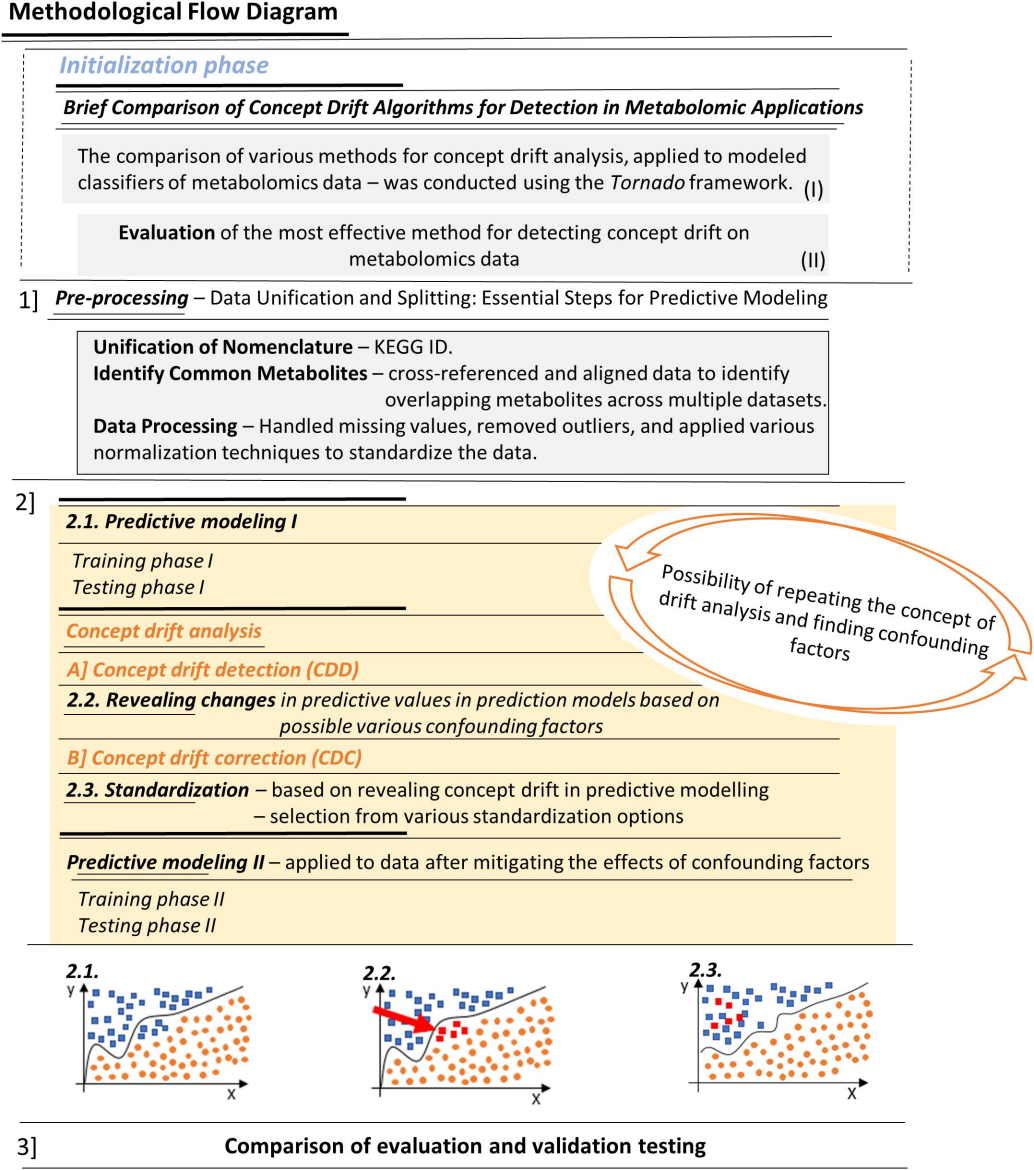
Methodological flow diagram: concept drift analysis and predictive modelling in metabolomics—from data pre-processing to concept drift detection, and model standardization.

Our study provides a detailed overview of the methodologies employed in this study, including data preparation, predictive modelling, and concept drift analysis, all aimed at enhancing the accuracy and reliability of metabolomic predictions. The methodological flow diagram (Fig. 1) outlines the key stages of the approach, which are explained in the following sections.

Each of these steps is critical to building robust predictive models, accounting for the dynamic nature of data and the influence of concept drift and confounding factors. The following sections describe each methodology in greater detail.

### 2.1 Initialization phase: a brief comparison of concept drift algorithms for detection in metabolomic applications

In the first step of the study, we utilized publicly available data from (Chu *et al.* 2021) and applied different CDD approaches for detecting concept drift in metabolomics predictive models. To achieve this, we compared several established methods for detecting concept drift using the Tornado framework (Pesaranghader *et al.* 2018), (https://github.com/alipsgh/tornado).

**Figure 2.**
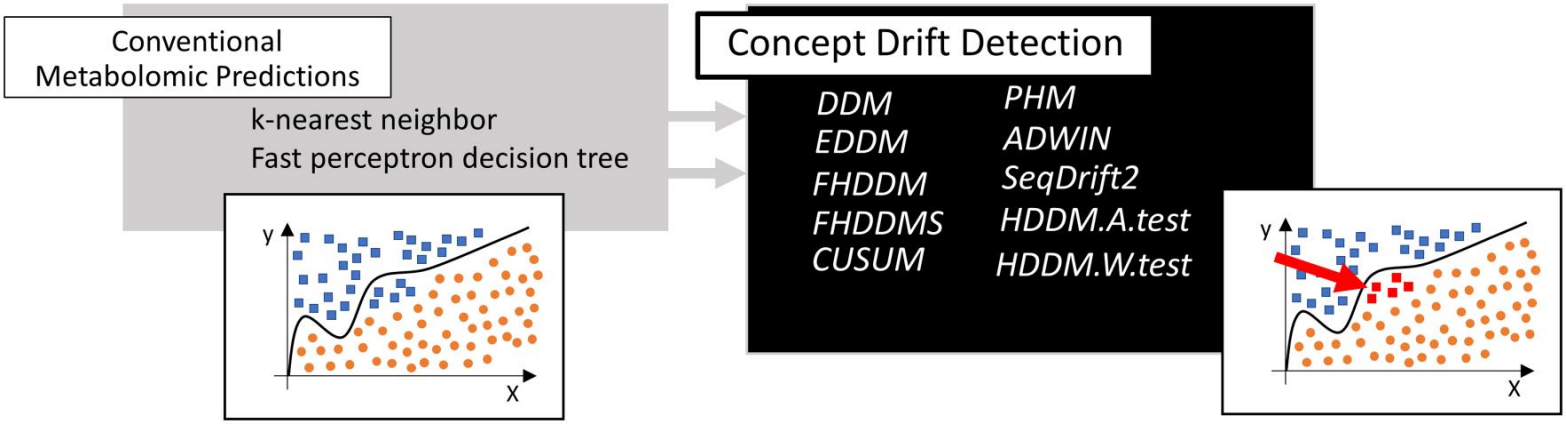
A summary of the applied model approaches and DDMs for evaluation of the most suitable candidate as a methodology for the CDD in metabolomic predictions using methods offered by Tornado (de Villiers *et al.* 2014, Pesaranghader *et al.* 2018). The left side of the figure represents conventional methods, ie learned classifiers. The right side of figure includes in the algorithms for the detection of concept drift in predictive models. These algorithms are able to estimate the concept drift based on the evenness of the data and point to a possible drift in the prediction, see the red arrow.

The comparison included ten different algorithms, with a focus on evaluating their effectiveness for detecting concept drift in prediction classifiers. As shown in Fig. 2, the left side of the diagram represents the conventional prediction models, such as k-nearest neighbour (kNN) (Abbad Ur Rehman *et al.* 2021) and fast perceptron decision tree (FPDT) (Bifet *et al.* 2010), which were used to model metabolomics data for gender classification. On the right side of the Fig. 2, the CDD algorithms are illustrated, which analyse the data to detect shifts in the relationship between the input features and the prediction outcomes (represented by the red arrow).

The methods compared in this study demonstrated varying degrees of assume concept drift, with the most effective algorithms providing meaningful insights into shifts in the data, thus informing the selection of the best-suited CDD techniques for use in metabolomic applications.

### 2.2 Unification of metabolomics datasets and clinical information

In this study, we utilized three datasets from human metabolomics studies(Chu *et al.* 2021, Li *et al.* 2022) to evaluate the performance of CDD methods. The first dataset originated from a study (Chu *et al.* 2021) by Chu *et al.* which investigates genetic differences affecting metabolite levels in a large cohort. The data, publicly available through the Human Functional Genomics Project (HFGP) (https://500fg-hfgp.bbmri.nl/). Public data from this study included 534 healthy individuals (240 males and 294 females) of Caucasian origin aged between 18 and 75 years (Table S1). Our study used normalized metabolite abundance levels from General Metabolomics (GM) and Nightingale Health/Brainshake (BM) metabolomic datasets by Chu *et al.* (2021) for the first phase of analysis (training and test data).

The second metabolomics data was taken from a study by Li *et al.* (2022) (such as validation data) which analysed metabolomic changes in cerebrospinal fluid (CSF) produced by acute endurance exercise. This dataset consisting of 19 healthy individuals (13 males and 6 females) aged between 18 and 37 years (Table S2) in three time points during exercise. Both studies have male and female participants and the goal of our prediction methods was to differentiate the gender in these studies.

The third dataset was obtained from Karlíková *et al* (2016), focusing on chronic myeloid leukaemia (CML) patients and the impact of tyrosine kinase inhibitor treatments on the metabolome. This dataset included 27 individuals: 15 healthy controls and 12 CML patients (14 males and 13 females), aged 29–76 years (Table S3). This dataset was used as validation dataset in this presented study to validate previously trained models and investigate the robustness of DDMs across different clinical scenarios.

To allow for the direct comparison of all studies specific metabolite features were unified for GM (training and testing), CSF and CML (validation) data across two studies by MetaboAnalyst (Xia *et al.* 2009). Missing values were filled with half of the minimum value using COVAIN (Sun and Weckwerth 2012) for pre-processed metabolite analysis. COVAIN defines outliers as measurements falling outside two standard deviations from the mean values for each condition of each compound.

Subsequently, several possible normalizations were implemented. All of them are user-friendly and available in a Google Colab notebook on GitHub (https://github.com/JanaSchwarzerova/SAPCDAMP). In this study, we tested all possible normalization variants during predictions, as described below in the Results section. The different normalization was calculated by different scaling taken from the study (van den Berg *et al.* 2006):

(1) $$\mathrm{Centering}\;{\bar{x}}_{ij}={x_{ij}-\bar{x}}_{i}$$

 

(2) $$\mathrm{Autoscaling}\;{\bar{x}}_{ij}=\frac{{x_{ij}-\bar{x}}_{i}}{s_{i}}$$

 

(3) $$\mathrm{Pareto}\;\mathrm{scaling}\;{\bar{x}}_{ij}=\frac{z_{ij}-{\bar{x}}_{i}}{{\bar{x}}_{i\;\max}-{\bar{x}}_{i\;\min}}$$

 

(4) $$\mathrm{Range}\;\mathrm{scaling}\;{\tilde{x}}_{ij}=\frac{{x_{ij}-\bar{x}}_{i}}{\sqrt{s_{i}}}$$

 

(5) $$\mathrm{Vast}\;\mathrm{scaling}\;{\bar{x}}_{ij}=\frac{{x_{ij}-\bar{x}}_{i}}{s_{i}}\;.\;\frac{{\overset{-}{x_{ij}}}_{i}}{s_{i}}$$

 

(6) $$\mathrm{Level}\;\mathrm{scaling}\;{\bar{x}}_{ij}=\frac{{x_{ij}-\bar{x}}_{i}}{{\bar{x}}_{i}}$$

 

(7) $$\mathrm{Log}\;\mathrm{transformation}\;{\bar{x}}_{ij}=\log_{10}(x_{ij})$$

 

(8) $$\mathrm{Power}\;\mathrm{transformation}\;{\bar{x}}_{ij}=\sqrt{x_{ij}}$$

**Figure 3.**
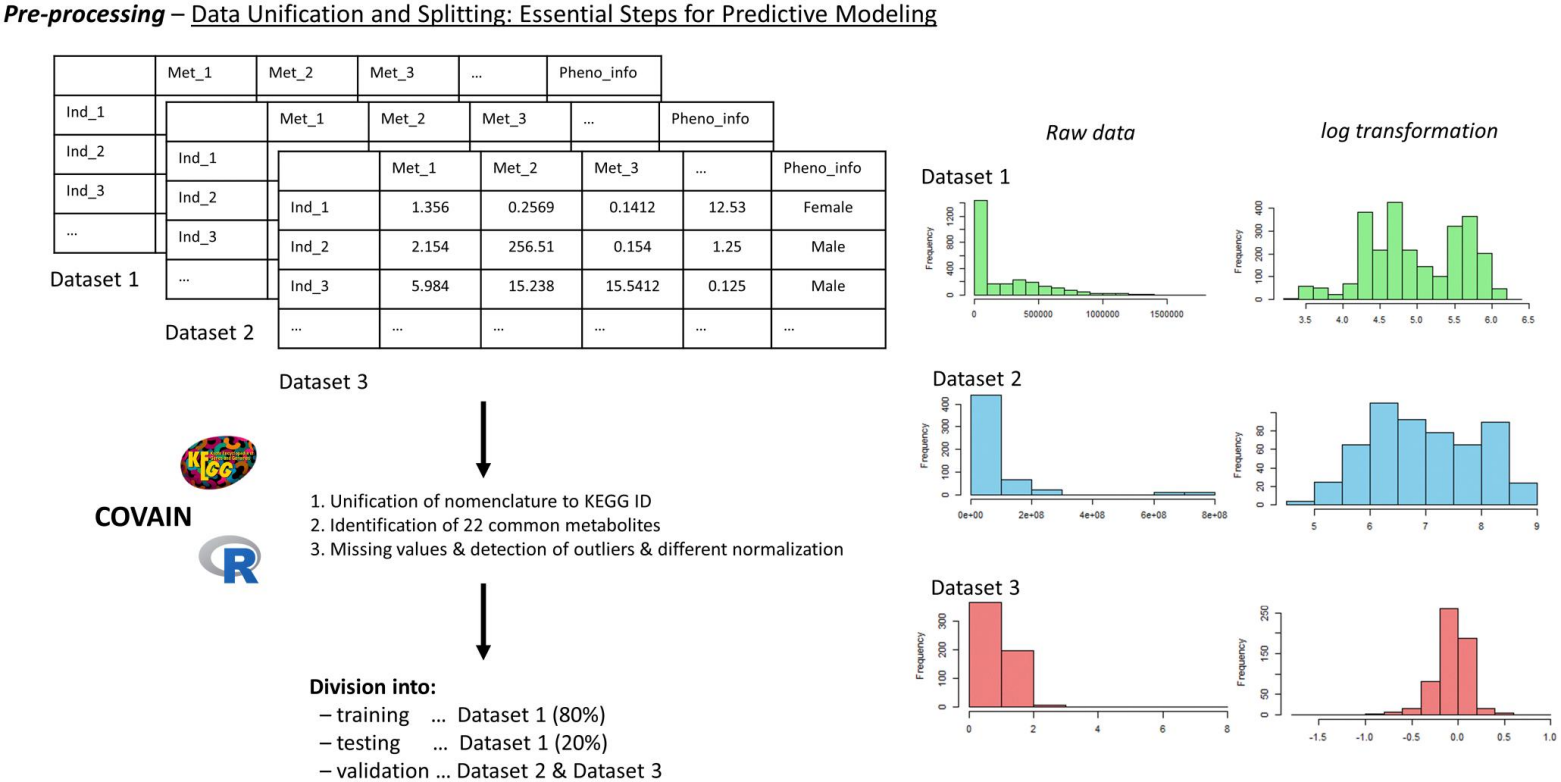
Pre-processing workflow for metabolomic data unification and splitting: The steps include unifying nomenclature to KEGG IDs, identifying 22 common metabolites across datasets, and pre-processing through handling missing values, detection of outliers and normalization. The data were divided into training (80% of Dataset 1), testing (20% of Dataset 1), and validation datasets (Datasets 2 and 3). The right part of the figure illustrates the distribution of individual datasets before and after applying normalization, with log-transformation chosen as an example.

The sample size ratio between training and test datasets was below 20/80 only in the case of the first dataset (GM data from Chu *et al.* 2021). The remaining two datasets (CSF and CML) were primarily used for validating models trained on the first dataset, as detailed in the GitHub repository and methodological overview of this unification and pre-processed part is shown in Fig. 3.

### 2.3 Overall design of the enhanced metabolomic prediction

The main goal of overall design for enhanced metabolomic predictions are shown in Fig. 3. The process for modelled enhanced metabolomics predictive classifiers (in our case determining the gender based on metabolite information) starts in modelling of classifiers of biological prediction or metabolomics prediction. Different predictive classifiers based on metabolomics were applied to determine the gender of individuals using metabolomic data.

These modelled classifiers were calculated by a variety of complex prediction methods such as kNN (Abbad Ur Rehman *et al.* 2021), Ridge Classifier (RC) (Aprit *et al.* 2013), support vector machine regression (SVR) (Brereton and Lloyd 2010), random forest (RF) (Chen *et al.* 2013, Kokla *et al.* 2019) implemented in python using scikit-learn (Pedregosa *et al.* 2011) and deep neural network (DNN) (Kopylov *et al.* 2021) using Keras (GitHub—keras-team/keras: DL for humans). It is shown that across a variety of complex methods better accuracy can be obtained but interpretability of models is lost. The parameters are optimized using different methods in training phase such as 10-cross validation (Ojala and Garriga 2010) or optimizer ADAM (Reddi *et al.* 2019) in neural network or in our case DNN. DNN modelling and architecture is described in Supplementary File S1.

Due to modelled predictive classifiers based on metabolomics data BM and CSF from the study by Chu *et al.* (2021), concept drift analysis was used to reveal the position of possible concept drift. The candidate positions in predictive models were associated with biological information from clinical trials [such as age or body mass index (BMI)] that were not used in the input data of predictive classifiers. At the end of our biological analysis, we also examine changes in predictions for CML patients. However, we acknowledge that these potential hypotheses require further validation in large prospective cohorts of patients, with multicentric clinical data on survival and responses to targeted therapy, immunotherapy, and chemotherapy.

The subsequent segmentation (Wind 1978) and standardization based on feature scaling of metabolomics data based on revealed confounding factors for a new model training will enable the enhanced metabolomic predictions with higher accuracy.

The standardization was performed based on the same principles as normalization using feature scaling, calculated with different scaling methods from the study (van den Berg *et al.* 2006), which are described in more detail above. However, *x* presents a matrix with two columns. The first column includes individual i metabolite and the second column is a binary classification determined on the basis of the threshold of the confounding factor detected, in our case age and BMI. The model derived for the study of Chu *et al.* was applied to data from the study (Li *et al.* 2022) and (Karlíková *et al.* 2016), then retrained using conceptual drift analysis to improve the prediction performance of classification, and potentially uncover additional hidden confounding factors in metabolomics.

## 3 Results and discussion

### 3.1 Comparison of methods for concept drift detection in application to metabolomics

**Figure 4.**
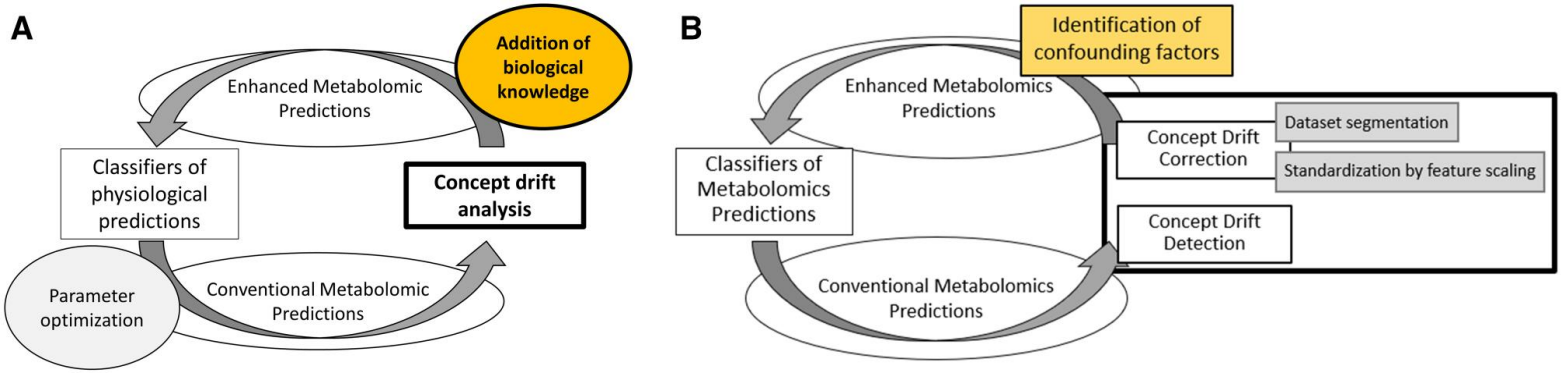
Presentation of the core methodology for developing enhanced metabolomic predictions using concept drift analysis. (A) The addition of biological knowledge (indicated by the yellow bubble) cannot be fully automated—these biological assumptions are usually based on the experience of experts. Therefore, our design pipeline is semi-automated. (B) The core methodology is expressed as an intermediate step to create the resulting semi-automated pipeline.

A comparison of different CDD methods using the Tornado framework (Pesaranghader *et al.* 2018), (https://github.com/alipsgh/tornado) applied on BM and GM data from Chu *et al.* (2021) reveals 59 concept drifts. Among the different methods tested, EDDM consistently detected the most (and same) drifts, as shown in Fig. 4. The DDM detector demonstrated similar performance, detecting concept drifts in metabolomics data as well.

Interestingly, the HDDM.A.test algorithm detected the most drifts for BM dataset when applied to the kNN classifier, compared to the other methods. However, EDDM only detected these drifts as at the warning level, which more likely indicates data drift, and does not necessarily translate into a change in the concept of predictive modelling. As results, we decided to focus on EDDM and DDM detectors for application to predictive metabolomic classifiers in the second phase of this study.

### 3.2 Establishment of a semi-automated pipeline

**Figure 5.**
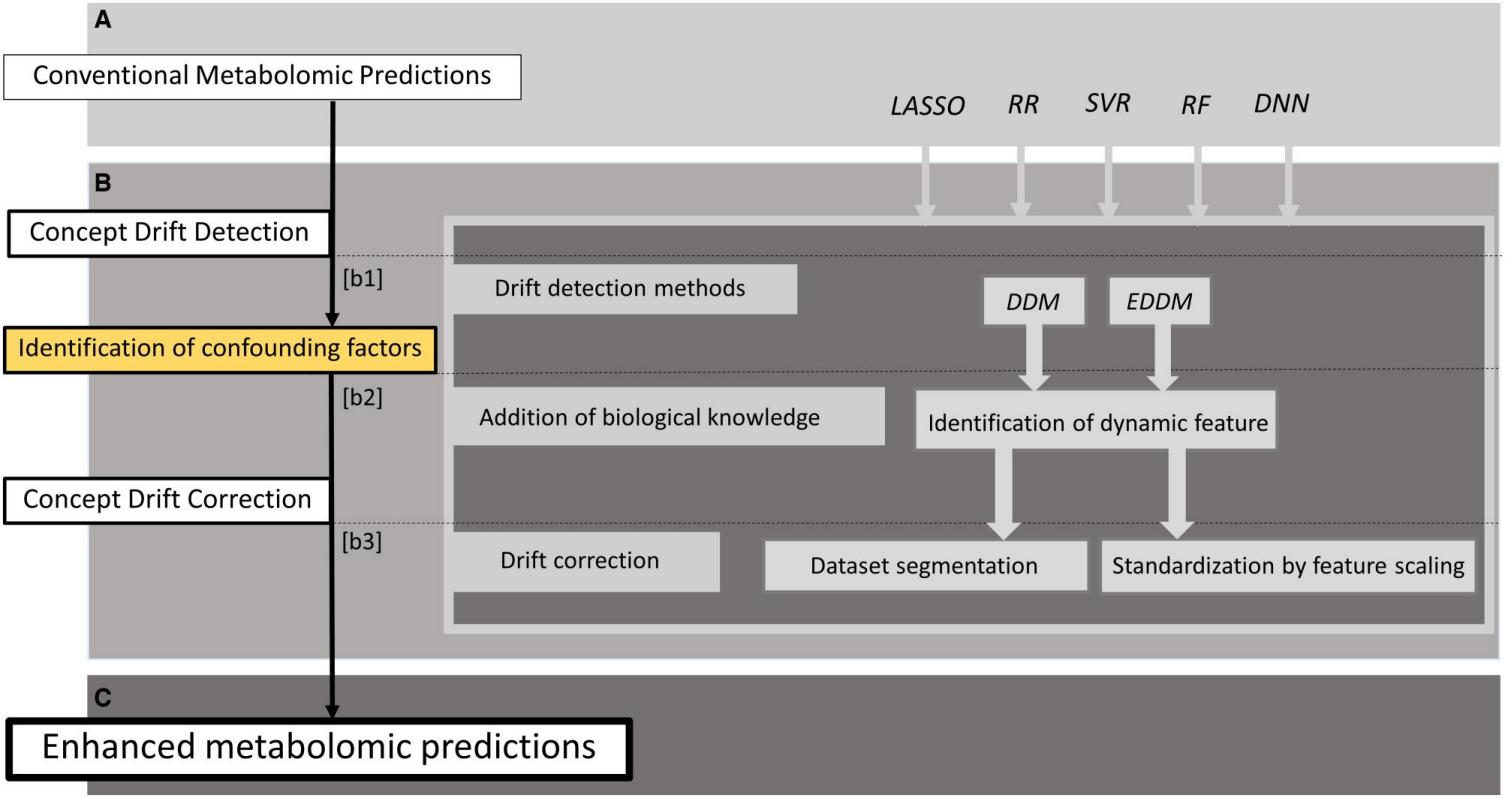
Semi-automated pipeline for enhanced metabolomic prediction classifiers based on concept drift analysis; (A) represents part of conventional metabolomics predictors using common approach, (B) is step of concept drift analysis which help to confirm confounding factors in metabolomic predictions, (C) presents a new enhanced metabolomic predictions improved based on the detection of confounding factors using concept drift analysis.

A semi-automated pipeline for enhanced metabolomic prediction was designed (Fig. 5) applied on unified datasets—GM (training and testing), CSF (validation) and CML (validation). A detailed description and instructions for users are available on GitHub: https://github.com/JanaSchwarzerova/SAPCDAMP. The whole pipeline is divided into three sections ([A], [B], [C]) at the end of which the evaluation parameters of individual prediction models are shown and summarized. Prior to Section A, conventional methods commonly used for visualization and classification in metabolomics, such as PCA was performed. This preliminary comparison with additional results is provided in the Supplementary File S2.

Section A represents selected conventional methods that are frequently used in predictive metabolomic modelling. The user can select from various predictive modeling methods, depending on the input dataset's dimensions and the predictability of the outputs. Table 2 summarizes the accuracy of different classifiers applied to datasets normalized using various scaling methods. Among the evaluated models, RF consistently demonstrated robust performance across most datasets and normalization techniques, achieving the highest accuracy of 0.75 on the CML dataset with centering and 0.76 on the CSF dataset with power transformation. RC exhibited strong accuracy in some cases, reaching a peak of 0.81 on the GM dataset with Pareto scaling. However, RC's performance on external validation datasets, such as the Li *et al.* dataset (Li *et al.* 2022), showed variability. In contrast, SVR demonstrated lower performance overall, with its best accuracy reaching only 0.34 on the CSF dataset with centering.

**Table 2.** Summary of accuracy for predictive modelling across different normalizations.

Normalization	Dataset	kNN	RC	SVR	RF	DNN
–	GM	0.58	0.78	0.05	0.68	0.45
–	CSF	0.60	0.61	0.25	0.73	0.69
–	CML	0.48	0.58	0.06	0.75	0.50
centering	GM	0.58	0.78	0.11	0.66	0.45
centering	CSF	0.60	0.61	0.34	0.75	0.69
centering	CML	0.48	0.58	0.07	0.70	0.50
autoscaling	GM	0.61	0.77	0.15	0.65	0.45
autoscaling	CSF	0.65	0.61	0.03	0.71	0.69
autoscaling	CML	0.63	0.57	0.16	0.70	0.50
range scaling	GM	0.61	0.74	0.15	0.65	0.45
range scaling	CSF	0.71	0.63	0.05	0.72	0.69
range scaling	CML	0.65	0.63	0.23	0.72	0.50
pareto scaling	GM	0.61	0.81	0.15	0.64	0.45
pareto scaling	CSF	0.65	0.63	0.03	0.75	0.69
pareto scaling	CML	0.63	0.67	0.16	0.70	0.50
vast scaling	GM	0.61	0.74	0.15	0.61	0.45
vast scaling	CSF	0.65	0.65	0.03	0.7	0.69
vast scaling	CML	0.63	0.62	0.16	0.67	0.50
level scaling	GM	0.54	0.75	0.16	0.66	0.45
level scaling	CSF	0.70	0.63	0.11	0.70	0.69
level scaling	CML	0.45	0.58	0.07	0.70	0.50
log transformation	GM	0.55	0.74	0.07	0.64	0.45
log transformation	CSF	0.76	0.6	0.24	0.75	0.69
log transformation	CML	0.40	0.67	0.08	0.70	0.50
power transformation	GM	0.63	0.73	0.11	0.69	0.45
power transformation	CSF	0.62	0.61	0.07	0.76	0.69
power transformation	CML	0.55	0.55	0.12	0.65	0.50

DNNs were also tested using an architecture adapted from our previous study (Schwarzerova *et al.* 2021). Detailed information on DNN modelling, including training, testing, and validation processes, is available in the Supplementary File S1. The DNN achieved a maximum accuracy of 0.69 on the CSF dataset under multiple normalization conditions, such as centering and range scaling. However, its accuracy dropped significantly to 0.31 when tested on the dataset from Li *et al.* (2022). Interestingly, the performance of the DNN was not affected by the choice of the normalization method, indicating that it is less sensitive to pre-processing variations. A similar, though less pronounced, trend was observed for RF. RF emerged as the most reliable classifier across datasets and normalization techniques, with ridge classifiers showing potential in specific cases. Despite their lower performance, DNNs and SVR could serve as supplementary approaches, depending on the application and dataset characteristics.

**Figure 6.**
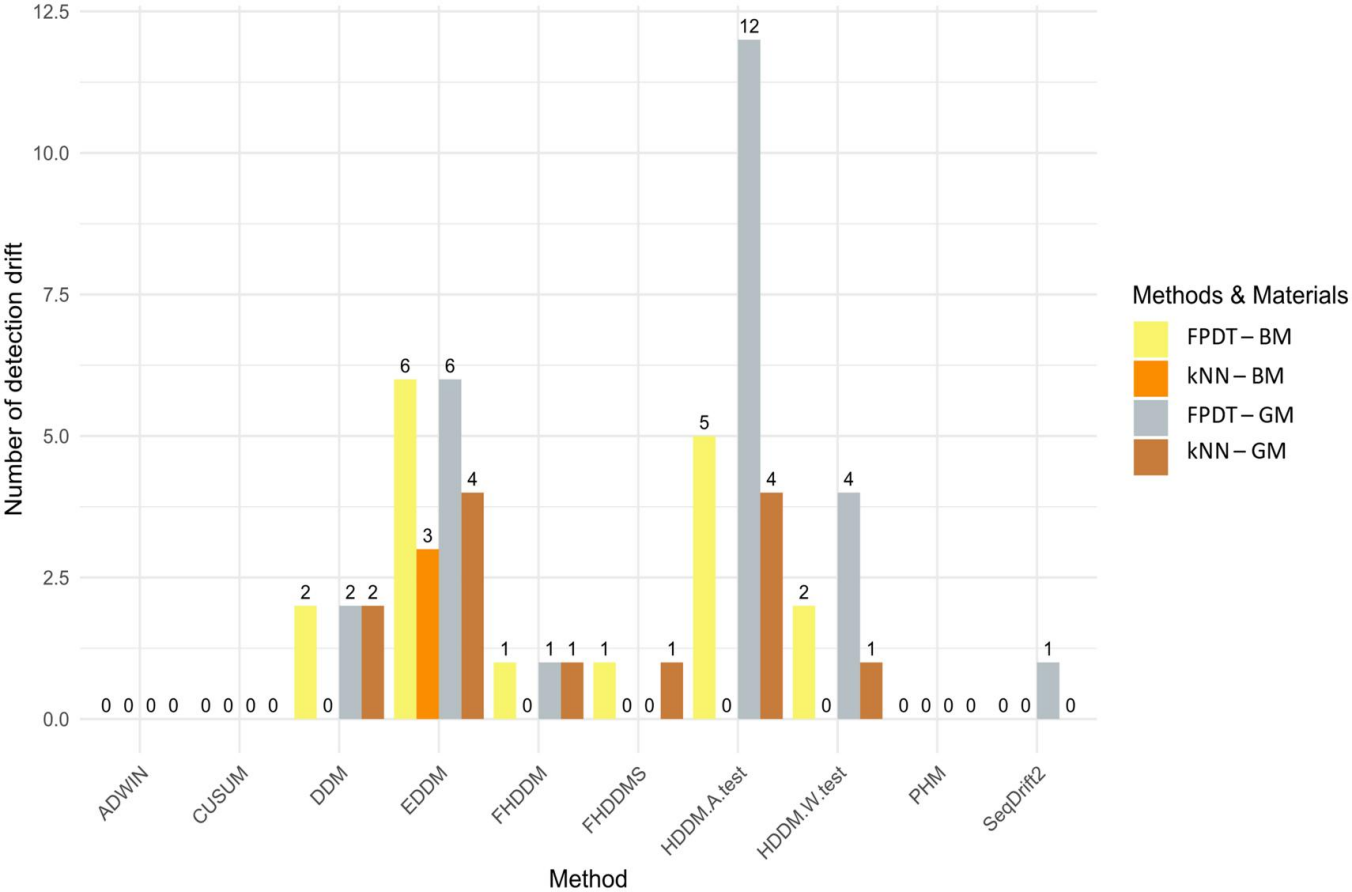
Summary of detected concept drift in different four predictive classifiers in metabolomic predictions. Yellow and orange colours represent BM metabolomics data and grey and brown colours represent GM metabolomics data from study (Chu *et al.* 2021).

Section B is structured into three distinct and systematically organized parts. As illustrated in Fig. 6, the DDM and EDDM methods were chosen as detection approaches based on prior results. In total, 10 concept drifts were identified: 8 in the RF classifier and 2 in the DNN model. For RF, concept drifts were observed across multiple normalization methods. With raw data, a single drift was detected using EDDM. Autoscaling, range scaling, power transformation, and log transformation all revealed the same concept drift in RF. In contrast, the DNN model exhibited only two detected drifts, both identified with log-transformed data using the EDDM method. Warnings of concept drift were also examined. RF produced warnings across all normalization techniques, with the highest frequency observed for raw data and range scaling. In comparison, warnings for DNN were restricted to raw data, autoscaling, and range scaling.

Interestingly, concept drifts in RF were often associated with individuals aged 21, 23, and 24 years. The mean age for DNN's warnings was ∼26 years, whereas the detected drifts in DNN corresponded to individuals aged 20. This observation is further explored in the subsequent chapter, which addresses the identification of confounding factors.

Based on this assumption, we tested two hypotheses [b3] included in our semi-automated pipeline for creating enhanced metabolomics classifiers. The first is based on proper segmentation [b3.1] of the input data. The segmentation is thought as a selection of a region of interest which was already described in (Schwarzerova *et al.* 2022). This approach is considered when an individual has a significant amount of data and can afford subject segmentation. Therefore, we mention it and keep this option available in our semi-automated pipeline. However, our main focus remains on scaling, which was performed based on the identified time threshold—in our case, 26 years.

Therefore, the second hypothesis relies on the transformation of the dataset using feature scaling [b3.2], thus, training and testing data is not lost as in segmentation techniques. The scaling was done based on the time-threshold found, in our case, i.e. 26 years.

**Figure 7.**
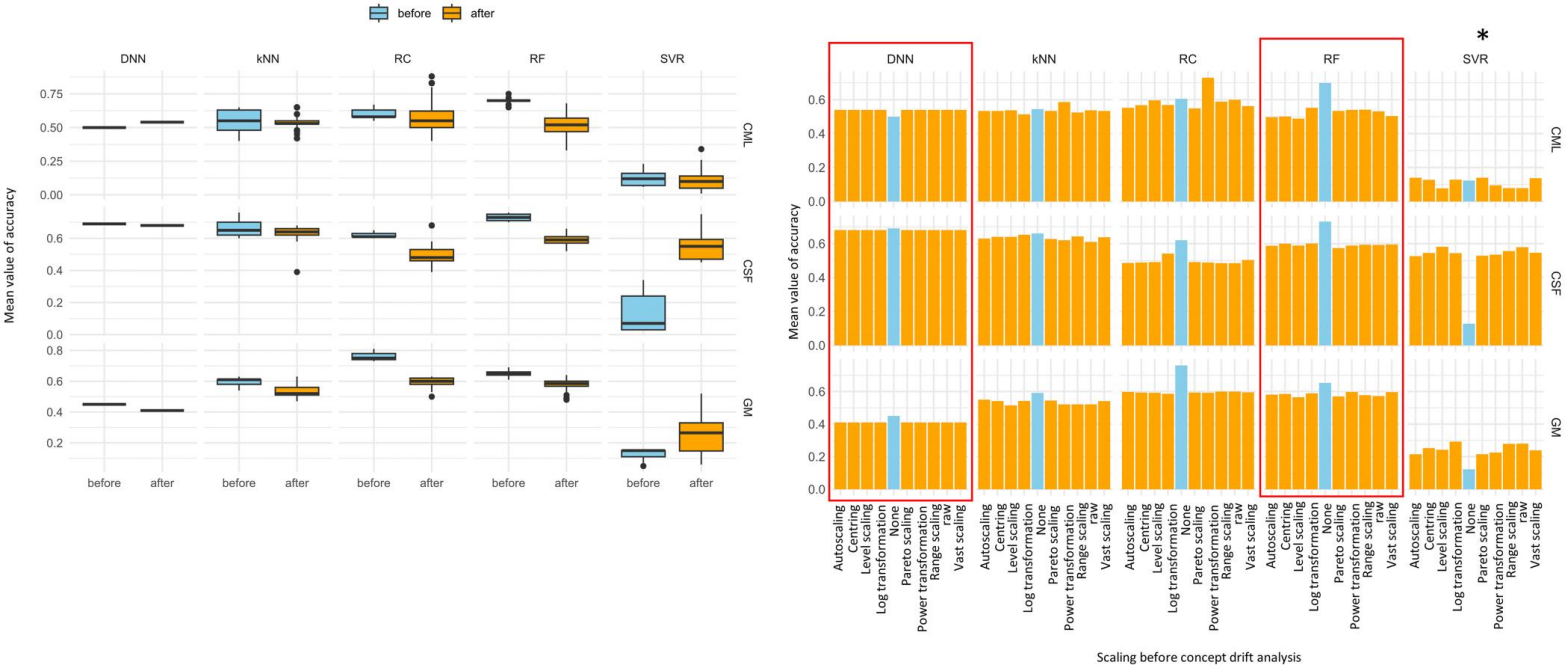
Summary of classifier accuracies before and after applying the concept drift analysis model, with results for different normalization techniques. On the left, box plots show the overall average accuracy across the various normalization methods. On the right, an example illustrates how input normalization can impact performance before (light blue) and after (orange) incorporating scaling with confusing factors. The red rectangle indicates the fully affected transmission due to scaling by the confounding factor.

Section C presents new modelling of classifiers using new data inputs to improve accuracy of classifiers. The overall summary of the accuracies of all modelled prediction classifiers shown, across different dataset, is in Fig. 7. The increase in classifier accuracy after concept drift analysis can be seen in three cases. The increase in accuracy within the segmentation correction is visible on the CSF data (test data). Some classifiers dropped their accuracy after dataset segmentation. However, this may be due to reducing the training and test dataset.

Based on our results, the conceptual analysis remains unsuitable for RF modelling. However, significant improvements in accuracy were observed with methods requiring substantial training data, particularly in the case of DNN models. When applying data normalization techniques—such as feature scaling for GM data and segmented data for CSF—there was a noticeable improvement in performance across multiple metrics (see Fig. 7). For instance, in GM data, the kNN accuracy rose from 0.58 to 0.61 with autoscaling and pareto scaling. For CSF data, pareto scaling and log transformations both resulted in improved model performances, with kNN accuracy increasing to 0.76. Notably, we also observed that no concept drift was detected in RF and DNN models after applying these new standardizations. It should also be emphasized that while transformation significantly enhanced model performance, the results were not uniform across all datasets and methods (see in Table S4).

### 3.3 Revealing confounding factors in human metabolomic predictions within phenotypic data

**Figure 8.**
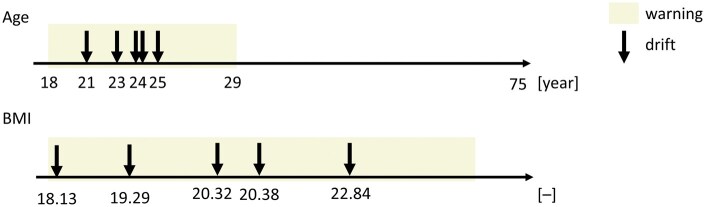
Visualizing the concept of drift analysis using possible confounding factors.

Using the concept of drift analysis in metabolomic predictive modelling also reveals the potential for detecting confounding factors in metabolomics. One well-known confounding factor, age, was confirmed in our analysis, with a significant shift detected around 26 years of age. However, warning levels ranged from 18 to 29 years within the DNN classifier (Fig. 8), indicating that the concept drift occurs in the adolescent period in relation to human predictions. Age is a well-established confounding factor in human metabolomics. Our findings reinforce the importance of accounting for age-related metabolic shifts, particularly in adolescence, where concept drift was most evident. The ability to correct for age-related effects enhances the reliability of predictive models in biomedical research.

On the other hand, BMI presents a more complex and less understood confounding factor. Our results did not provide a decisive conclusion regarding BMI as confounding factors in metabolomic predictions. However, both detected drifts and the warning level were more pronounced at lower BMI levels, suggesting a potential link between malnutrition and metabolic variability. This raises important questions regarding whether malnutrition introduces greater confounding effects than obesity in metabolomics-based predictions. Large population-based metabolomics studies indicate that metabolic BMI (mBMI) can be used to define obesity and identify hidden risk for metabolic disorders independently of measured BMI. Differences between BMI and mBMI allow for the identification of individuals with similar BMI but distinct metabolic health profiles and phenotypes. This observation may explain why BMI, unlike age, exhibits a broader range of influence as a confounding factor when analysed through concept drift methods (Beyene *et al.* 2023).

Regarding cancer patients, our analysis revealed a 58% false positive rate, suggesting the presence of an unaccounted-for confounding factor. However, due to the lack of a consistent dataset, this hypothesis remains unconfirmed. Adjustments to the prediction model incorporating adolescent age as a contributing factor led to performance improvements, highlighting the need for further exploration of confounding factors beyond known variables. However, our outputs look promising and open up new possibilities for further studies.

### 3.4 Revealing confounding factors in human metabolomic predictions within metabolomic data

Beyond phenotypic factors such as age and BMI, metabolite-specific confounding factors were also investigated. We hypothesized that specific metabolites play significant roles in metabolic regulation, disease progression, and predictive model performance. Concept drift analysis enabled us to assess whether individual metabolites serve as confounding factors in predictive models.

The following metabolites were examined: amino acids (L-Alanine, L-Arginine, Glutamine, Serine, Methionine, L-Tryptophan, Phenylalanine, L-Tyrosine, Histidine, L-Asparagine, L-Threonine), fatty acids and derivatives (Arachidonic acid, Taurine), nucleotides and related compounds (Gluconic acid, Inosine, Uridine), and other metabolites (Citrulline, Creatinine, Hippuric acid, Xanthosine). Among these, specific hypotheses were tested: Creatinine was examined as a potential confounding factor, as it serves as a pathological marker, particularly in contexts related to muscle metabolism and exercise.

**Figure 9.**
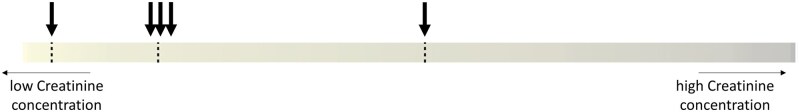
Visualizing the concept of drift analysis by grey-scale representing Creatinine concentration with highlighted CDD.

Based on the visual analysis of the Creatinine concentration and the highlighted CDD in Fig. 9, we can draw some important conclusions about the role of Creatinine as a potential confounding factor in metabolic predictions. The colour scale representation effectively illustrates the variations in Creatinine levels across different data points, with specific concentrations highlighted as potentially influencing the predictive model outcomes. This suggests that Creatinine, particularly in its role as a pathological marker for muscle metabolism and exercise, may act as a confounder in metabolomic studies. The concept drift analysis further supports this hypothesis by indicating shifts in its relationship with other metabolites over time, potentially affecting the model's performance and accuracy.

These findings underscore the importance of considering individual metabolites like Creatinine in metabolic studies, especially when developing predictive models that might be impacted by shifts in metabolic patterns due to factors such as exercise, disease progression, or aging. Therefore, it is crucial to incorporate such confounding factors into the model development process to ensure more reliable and accurate predictions in metabolomics studies.

## 4 Conclusions

This study designed and demonstrated enhanced predictive modelling approaches using concept drift analysis for metabolomic predictions. The presented semi-automated pipeline opens new possibilities for advanced modelling, highlighting the importance of concept drift analysis in identifying confounding factors that may bias predictive models. Here we show, that detecting confounding factors may help for model correction and improves metabolomic predictions. By incorporating concept drift analysis into metabolomics, we are able to recognize fluctuations in metabolite levels that could otherwise go unnoticed, revealing hidden biases in the data. The visual representation of these variations, particularly in Creatinine concentrations, demonstrates abrupt transitions that suggest shifts in underlying biological or technical factors. These transitions align with changes in patient age, BMI, and other cohort characteristics, highlighting Creatinine as a potential confounding factor. Such shifts could be driven by several factors, including sample processing inconsistencies, cohort variations, or external environmental influences, all of which may impact the predictive accuracy of models. This underscores the need for dynamic adjustments to predictive models to account for these variations over time.

By integrating CDD into metabolomic studies, we gain deeper insights into previously unrecognized confounding factors, which can ultimately enhance the robustness and reliability of models. This approach not only identifies known confounders but also provides a systematic method for detecting emerging factors that may influence predictions. The findings from this study emphasize the importance of refining metabolomics-based predictive models by systematically accounting for both established and newly identified confounding factors. This study demonstrates that adjusting models to remove or confirm these confounders can improve the interpretation of large datasets, leading to more accurate predictions and better-informed research into new biomarkers and metabolic health.

The proposed procedure is arranged into a semi-automated pipeline which is available on github.com/JanaSchwarzerova/SAPCDAMP. Importantly, the presented methods revealed confounding factors in metabolomic predictions such as age and other possible factors associated with a general metabolic status. While these factors might find utility as advanced predictive approaches for research in metabolomics or lipidomics, they can help to unravel approaches in the pursuit for new biomarkers or markers. As our study shows, the prediction models can be re-optimized to remove or confirm other important confounding factors and thus help with removing the biological bias and better interpretation of large datasets.

## Supplementary Material

vbaf073_Supplementary_Data

## Data Availability

There are no new data associated with this article.
