# Supplementary material for: Enhanced metabolomic predictions using concept drift analysis: identification and correction of confounding factors
Source: Bioinform Adv. 2025 Apr 4;5(1):vbaf073. doi: 10.1093/bioadv/vbaf073 (PMC12037104; doi:10.1093/bioadv/vbaf073)
Supplement: vbaf073_Supplementary_Data [file vbaf073_supplementary_data.zip › Briefly_view_into_DNN.pdf]

Overview of the Deep Neural Network Architecture for Metabolite Analysis using in https://github.com/JanaSchwarzerova/SAPCDAMP

Jana Schwarzerová

This deep neural network (DNN) architecture was designed to classify metabolite data, leveraging its high-dimensional and often non-linear nature. The model has been optimized for feature representation and prediction accuracy by incorporating fully connected layers and appropriate activation functions.

The approach has been validated and published in the IEEE BIBM proceedings (DOI: 10.1109/BIBM52615.2021.9669418).

Architecture and Rationale

Input Layer:

The input dimension of the model is tailored to metabolite datasets, which are typically high-dimensional (e.g., metabolomics data often have hundreds or thousands of features). Here, the input size reflects a preprocessed subset of the most informative features. This preprocessing step ensures the model can effectively handle noisy and redundant data.

Hidden Layers:

Fully Connected Layers:

The model includes multiple dense layers with 150, 50, and 80 neurons. These layers progressively learn abstract feature representations, capturing complex, non-linear relationships among metabolites.

The ReLU activation function in these layers prevents vanishing gradients and introduces non-linearity, which is crucial for learning intricate patterns in metabolomic data.

By stacking layers, the model can capture hierarchical information, starting from low-level features to high-level abstractions related to metabolic pathways and interactions.

Softmax Layers:

Intermediate layers with Softmax activation are incorporated to encourage probabilistic interpretations and improve feature importance in classification tasks. This is particularly useful for metabolic studies, where probabilistic outputs help infer the likelihood of specific metabolite pathways or groups being relevant to the target classification.

Output Layer:

The final output layer uses a Sigmoid activation function, appropriate for binary classification tasks, such as distinguishing between healthy and diseased states or between two metabolite conditions.

The use of *categorical_crossentropy* allows the model to perform well in multi-class setups when combined with one-hot encoding of the target variable.

This supplementary text has been reviewed and refined with the assistance of artificial intelligence, which contributed to improving its clarity, coherence, and overall quality.

The effectiveness of Fully Connected Layers for metabolite analysis

The choice of fully connected layers in the architecture for metabolite analysis is deliberate, addressing the specific challenges and characteristics of metabolomic data. These layers provide several advantages that align well with the needs of this domain:

Feature Importance and Interpretability

One common criticism of deep learning models is their "black box" nature, where decision-making processes are opaque. However, in the context of metabolomics, this issue can be mitigated through techniques like SHAP (SHapley Additive exPlanations) or other feature importance methods. These tools can identify which metabolites contribute most to the model's predictions, offering biologically meaningful insights. For example, key metabolites identified as important could point to specific pathways or biomarkers relevant to the studied condition. Such interpretability is critical for bridging the gap between computational models and their application in biological research.

Capturing Non-Linear Relationships

The interactions between metabolites are inherently complex and rarely linear. Factors such as enzymatic activities, metabolic pathway dependencies, and environmental influences create a web of non-linear interactions. Fully connected layers, with their ability to learn intricate relationships through weight optimization and non-linear activation functions like ReLU, are well-suited to this challenge. Unlike traditional statistical approaches that may assume linearity or simpler dependencies, DNNs can uncover patterns that would otherwise remain hidden, making them invaluable for metabolomics.

Dimensionality Reduction and Feature Abstraction

Metabolomic datasets often exhibit high dimensionality, with thousands of metabolites measured across a relatively small number of samples. This "curse of dimensionality" can make it difficult to identify meaningful patterns or to avoid overfitting. Fully connected layers naturally perform dimensionality reduction by learning compressed, abstract representations of the data in their intermediate layers. These representations capture the most relevant features for the task at hand, effectively reducing noise and redundancy. This capability ensures that the model is not only efficient but also generalizable to unseen data.

Publication and Validation

This architecture has been validated through rigorous experimentation and published in the IEEE International Conference on Bioinformatics and Biomedicine (BIBM) 2021, DOI: 10.1109/BIBM52615.2021.9669418. The study demonstrated the model’s effectiveness in classifying metabolic datasets with high accuracy, precision, and recall, showcasing its potential for practical applications in metabolomics research.

The results highlight the DNN's ability to not only provide robust predictions but also offer insights into the metabolic pathways underlying the classification tasks, paving the way for future studies and applications in marker discovery and personalized medicine.

This supplementary text has been reviewed and refined with the assistance of artificial intelligence, which contributed to improving its clarity, coherence, and overall quality.
