# Supplementary material for: Enhanced metabolomic predictions using concept drift analysis: identification and correction of confounding factors
Source: Bioinform Adv. 2025 Apr 4;5(1):vbaf073. doi: 10.1093/bioadv/vbaf073 (PMC12037104; doi:10.1093/bioadv/vbaf073)
Supplement: vbaf073_Supplementary_Data [file vbaf073_supplementary_data.zip › PCA_ vs_Concept_drift_analysis.pdf]

PCA Analysis vs Concept drift analysis of Metabolomics Data:

Principal Component Analysis (PCA) was performed on a combined metabolomics dataset comprising three different sources: (Chu *et al.*, 2021), (Karlíková *et al.*, 2016), and (Li *et al.*, 2022a). The analysis aimed to reduce the dimensionality of the data and visualize any potential patterns or groupings related to variables such as age and gender. The dataset was cleaned by removing non-relevant columns (ID, and age) for the purpose of analysis. Afterward, the data was standardized (scaled) to ensure that variables with different units or magnitudes would not dominate the analysis.

The PCA was conducted on the scaled dataset, excluding age and gender columns from the analysis to focus on the metabolite data. The first two principal components (PCA1 and PCA2) were then extracted, capturing the largest variance in the data The PCA plot (Figure S1) shows the distribution of samples based on their first two principal components (PCA1 and PCA2), with points colored according to age.

The plot () reveals possible age-related clustering in the data, which may reflect metabolic changes associated with age. Several individual data points (identified by ID labels) are also annotated on the plot to highlight specific observations.

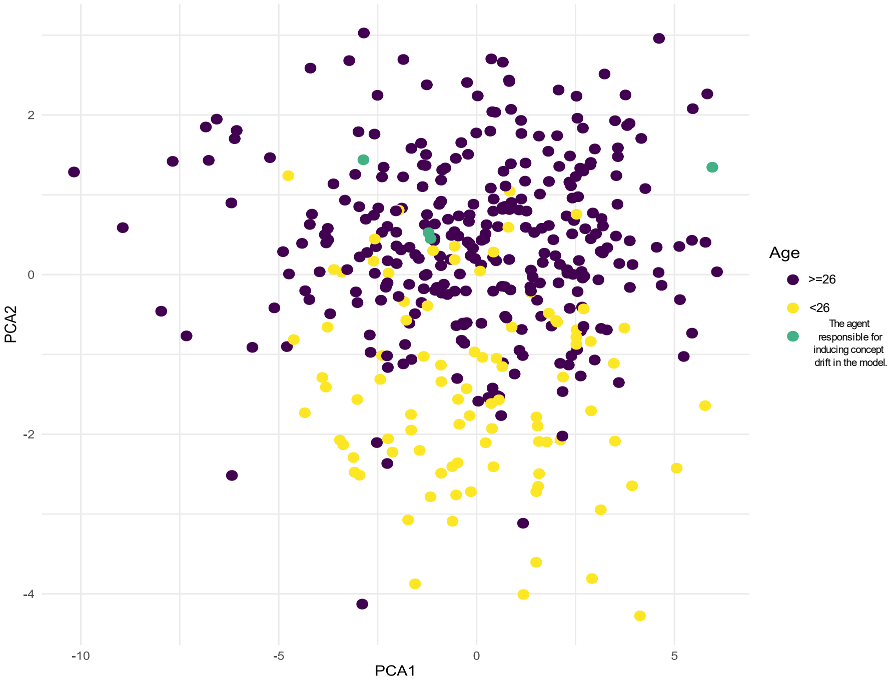

Figure S1: PCA - Visualization of metabolomics data by age, green colour represents samples playing a significant role in the concept of drift detection
